# State Medical Association of Texas, Dallas Meeting, May 6, 7, 8, and 9, 1902

**Published:** 1902-06

**Authors:** 


### STATE MEDICAL ASSOCIATION OF TEXAS, DALLAS MEETING, MAY 6, 7, 8, AND 9, 1902.

### THE REORGANIZATION MUDDLE.

Some months ago President Taylor Hudson appointed Drs. J. T. Wilson, J. E. Gilcreest, H. A. West, S. C. Red and R. F. Miller as a committee on revision of the Constitution and By-Laws of the State Association. These gentlemen held a meeting at Dallas in February of the present year and duly formulated a report. Later, as the result of considerable correspondence between Dr. West and Dr. George H. Simmons, Secretary of the American Medical Association, it developed that the adoption of this report would not be acceptable to the American Medical Association, and would not result in giving the State society a Constitution and by-laws similar to that likely to be adopted by the other States and Territories; consequently, at a second meeting, attended, unfortunately, by only two of the five members (Drs. West and Red), certain radical changes were inserted to make the report conform with the recommendations made by the reorganization committee of the American Medical Association. This second, or revised report, with an explanatory note, was printed and sent to the various affiliated societies and to the individual members of the State Association.

Dr. West disclaims all responsibility for the misleading statement of contents printed on the cover of this pamphlet.

When the Dallas meeting was called to order, it was found that the other three members of the committee favored the original report, while the remaining two favored the revised report, and, that no compromise was possible. Consequently, upon motion, the entire matter was referred to a body made up of the drafters of the two reports, the President of the Association, the delegates from the affiliated societies, and a representative from each of the congresional districts. After an entire day's deliberation, the new committee also failed to reach an agreement. Therefore, when the matter was again presented to the executive session on the following morning, it was decided by a practically unanimous vote to postpone final action until the 1903 meeting. In the meantime, the secretary has been instructed to have both of the reports in question printed and a copy of each sent to every member of the Association.

The reports differ in but one or two essential particulars. These points of difference, however, are of vital importance. Briefly stated, they are as follows:

The majority or unpublished report provides that (a) affiliated county and district societies are entitled to representation in the legislative branch of the State Society, the number of representatives being determined by their total membership; (b) the members of affiliated societies are eligible to membership in the State Society, but it is optional with them as to whether they join or not; (c) the delegate body of the State Society may re-district the State with the consent of the interested local societies.

The minority report requires that (a) the State be divided into fifteen districts, the distribution of the medical population being taken as the basis of division; (b) the membership of the county societies of each district shall constitute the district society, and the membership of the district societies taken together shall in turn constitute the State Society.

This is by far the most important problem that has ever been presented to our State organization for solution. Its extreme importance merits for it careful study by every physician in the State who has the interests of the profession at heart.

It will be observed that the plan proposed by the majority of the committee differs in no essential particular from the plan upon which the State Association is now organized. We are unable to discern in it any provision that will tend to strengthen or improve the present flimsy organization, or to increase its membership. It gives the State organization absolutely no authority over the county and district societies. It allows the members of the lower organization representation in the management of the affairs of the State Association, whether they have elected to be its members and supporters or not. Could anything be more absurd? What can be said in support of a proposition to admit outsiders to take a hand in the management of the affairs of an organization to which they contribute neither moral nor financial support?

The minority report, on the other hand, outlines a plan of organization precisely similar to that recommended by the Committee on Reorganization of the American Medical Association. By the adoption of this report, the county societies in each district are united to form the district societies, and the district societies are in turn united to form the State Association. The per capita tax necesary to support this organization will, of course, for obvious reasons, grow steadily less year by year as the county societies multiply and their membership increases. The plan is not a new one. It contains no novel or untried features. Every organization not purely local in its character, is constituted in some such manner. The organic law of churches, fraternal orders and the like present these same features modified in non-essential particulars. The objection that the tax necessary to support the State and district associations will prove too great a burden, might be made to apply with equal force in the case of every organization which reaches beyond a single town or county. To be sure it would be cheaper to belong to an isolated society, having no connection with any other. To carry the argument a step further, it would be cheaper not to belong at all. After all, it is merely a question of organization or no organization. The great benefits, both to the public and to the organization, of uniting the profession through every part of the State, will surely be worth many times the cost of its support.

W. B. Russ.

---

The Texas Medical Journal, be it always understood, stands for the profession at large and for no section of the State and no faction, and for the best interests of the State Medical Association, which interests it has always championed and defended. I take occasion to differ with my esteemed colleague when he says that, "after an entire day's deliberation the new committee also failed to reach an agreement." The committee took the two reports or drafts of a proposed Constitution which came to be known respectively as the "majority report" and the "minority report," and from them carefully and laboriously worked out a new Constitution, which it was hoped and believed would meet the views of the Association, and reconcile the differences between the authors of the two reports. The new draft was gone over carefully, and every feature was considered and discussed before adoption. It was adopted section by section, and finally as a whole, with one dissenting vote, as I remember (and I was on the committee—as well as Dr. Russ). Dr. M. M. Smith, who was the secretary of the committee, and Dr. Bacon Saunders, who was chairman, agree with me on this point. Dr. Smith was instructed to present the new draft of a Constitution to the Association the next morning as the unanimous report of the committee. Dr. Russ has told its fate. It is this report, however, which is to be published—along with the original (West and Red) "minority report," and not the Wilson, or "majority report," referred to. The minority report (West and Red's) appeared in the *Red Back* for May. At the time it was published, I was under the impresion that it was the report of the full committee, and did not know that the majority of that committee had no voice in it, and repudiated it, nor that they would submit, as they did, a "majority report."

The question seems to be: To district or not to district the

State? It is argued by those who favored the Wilson or majority plan, that the several large district associations are the accretion around small foci of organizations, and represent years of persistent labor; that the membership is drawn not entirely from the immediate vicinity, but from a large and undefined area, from points very remote from each other, and that to divide the State into geographical sections or districts would cut off a very large part of their membership, leaving them in a district where there is no organization; and that there is no way in which the members thus cut off, or the members of the profession who do not belong to any society, can be made to organize. Take the North Texas Medical Society, whose membership, I am informed, exceeds that of the State Medical Association. It meets semi-annually at points in North Texas accessible to its members,—many of whom live in the Panhandle country, where there is no organization. Take the E. T. Medico-Chirurgical Society. It meets semi-annually at Palestine, the most central point. The proposed districting would rob these—and also the Austin District, the S. W. Texas District, the Central Texas District and all other district societies of a portion of their membership, and would leave those thus cut off without a nucleus of organization around which they could rally and reform. Take the Victoria section of country. It is somewhat remote from any organization and, although there are eleven practicing physicians at Victoria, as many or more at Cuero, Port Lavaca and other nearby points—some of whom belong to the San Antonio and some to the Austin Society—Dr. Crouse, of Victoria, says they have never been able to effect an organization. And yet the Victoria men, and others from that section, favor the plan of putting them in a district by themselves. I fail to see how this would be of any assistance in effecting an organization, while it would cut them off from the existing organizations at San Antonio and Houston and in other districts.

I regret to see the tendency of this dissension. It is shaping into a sectional war, and I much fear that without good statesmanship and a spirit of conservatism, it will lead to an open rupture and a secession of North Texas from the State Association. The membership of the already existing societies is very dear to the organizers and they will not give up a large share of it without a fight. At San Antonio next April I hope a spirit of tolerance and good will, a spirit of concession and compromise, and above all, a spirit of fairness and justice will prevail, and that all sectional interests, jealousies and differences will be sunk in the larger interest of the State Association and the welfare of the whole profession of the State. We want unity and harmony. We want to organize from the ground up, and there is a way to do it. Let us be patient and careful, and find that way.

